# Exploring the Scientific Rationality of the Phenomenon of “Different Dosage Forms of the Same Prescription” of Chinese Proprietary Medicine Based on Biopharmaceutical Properties of Powder and Pill of Chuanxiong Chatiao Prescription

**DOI:** 10.3389/fphar.2022.893552

**Published:** 2022-06-09

**Authors:** Zhong-huan Qu, Lin Liu, Xiao-fei Zhang, Dong-yan Guo, Bing-tao Zhai, Jun-bo Zou, Ya-jun Shi

**Affiliations:** ^1^ Pharmacy College, Shaanxi University of Chinese Medicine, Xianyang, China; ^2^ Shangluo Hospital of Traditional Chinese Medicine, Shangluo, China

**Keywords:** Chuanxiong Chatiao prescription, different dosage forms for the same prescription, tetramethylpyrazine, ferulic acid, nodakenin, isoimperatorin

## Abstract

**Background:** The 2020 edition of the Pharmacopoeia of the People’s Republic of China (Chinese Pharmacopoeia 2020 edition) has 255 Chinese prescriptions with different dosage forms, accounting for 21.09% of the total prescriptions (1,209) in Chinese Pharmacopoeia 2020 edition. However, the scientific rationality of the phenomenon of “Different Dosage Forms of the Same Prescription” of Chinese proprietary medicine has been less explored. Based on the dosage form theory of “components in pills release slowly and take effect in slow-acting manner, while in powders release quickly and take effect in fast-acting way,” we provided the *in vitro* dissolution experiment and *in vivo* pharmacokinetics of Chuanxiong Chatiao powders and pills in order to rationalize the phenomenon of “Different Dosage Forms of the Same Prescription” of Chuanxiong Chatiao prescription.

**Materials and Methods:** Chuanxiong Chatiao powders and pills were prepared in the laboratory referring to the preparation methods in the Chinese Pharmacopoeia 2020 edition, and the contents of tetramethylpyrazine, ferulic acid, nodakenin, and isoimperatorin were determined by the external standard method. We measured the *in vitro* dissolution of four analytes of Chuanxiong Chatiao powders and pills according to the second method for dissolution determination (paddle method) in the Chinese Pharmacopoeia 2020 edition, and their corresponding contents in each sampling point were determined by LC-MS/MS. We also provided a pharmacokinetic study of Chuanxiong Chatiao powders and pills. Six female domestic rabbits were divided into two groups (powder and pill groups) and given Chuanxiong Chatiao powders and pills (9.85 g/kg) by surgical administration separately. Blood samples were collected at 5, 15, 30, 45, 60, 90, 120, 150, 180, 240, 360, 480, 720, and 1,440 min after drug administration to measure the plasma concentration of the four analytes by LC-MS/MS.

**Results:** The results of *in vitro* dissolution experiment showed that the dissolution rate of four analytes in the powder group was greater than that of the pill group. However, the solubilities of tetramethylpyrazine and isoimperatorin were very low in the powder and pill, which may be related to their low solubility properties. The results of the *in vivo* pharmacokinetic study of Chuanxiong Chatiao powders and pills showed that *T*_max_ (h) of ferulic acid and nodakenin in the powder group was 0.420 and 0.053 times that of the pill group and *t*_1/2_ (h) of ferulic acid, nodakenin, and isoimperatorin of the powder group was 0.910, 0.262, and 0.661 times that of the pill group, respectively.

**Conclusion:** The *in vitro* dissolution rate and *in vivo* pharmacokinetic parameters of four analytes in CXCTF could partly explain the scientific rationality of the classic theory of “丸者缓也, 散者散也” as in Chinese, which is helpful for providing a basis for the comparison of subsequent dosage forms. The results of our studies also suggest the complexity of the design of dosage forms of Chinese proprietary medicines and imply that we should pay more attention to the scientific rationality of the phenomenon of “Different Dosage Forms of the Same Prescription.”

## 1 Introduction

When Chinese proprietary medicines (CPM) are applied to the clinical treatment of diseases, they can be made into various dosage forms according to their physical and chemical properties and clinical needs, such as decoctions, pills, powders, tablets, granules, plasters, solutions, syrups, tinctures. In recent years, with the improvement of modern technology and the discovery of many new excipients, the progress of dosage form reform of CPM has been accelerated, which contributes to the diversification and modernization of the dosage form of CPM (Lu Xi and Yao, 2014; Nie et al., 2021) and the gush of a large number of prescriptions with different dosage forms and the same main functions. According to the statistics of our group, the Chinese Pharmacopoeia 2020 edition has 255 Chinese prescriptions with different dosage forms, accounting for 21.09% of the total prescriptions (1,209), including 166 prescriptions with two different dosage forms, 55 prescriptions with three dosage forms, 21 prescriptions with four dosage forms, and 13 prescriptions with the number of dosage form more than or equal to 5. The statistical results are shown in Table 1. These data show that prescriptions with the same formula composition but different dosage forms are abundant in the Chinese Pharmacopoeia 2020 edition. It is necessary to study deeply how to reasonably choose CPM with “Different Dosage Forms of the Same Prescription.”

**TABLE 1:** Statistics on prescriptions with the phenomenon of “Different Dosage Forms of the Same Prescription” in Chinese Pharmacopoeia 2020 edition.

Number of dosage forms	Number of prescriptions	Percentage (%)	Percentage of prescriptions with different dosage forms (%)
1	954	78.91	—
2	166	13.73	21.09
3	55	4.55	
4	21	1.74	
≥5	13	1.08	

Chuanxiong Chatiao prescription (CXCTP) is a classic formula for the treatment of migraine headaches (Li, Zhang, Sun, Fang, and Shou, 2020), which was first recorded in the form of powders in *Taiping Huimin Hejiju Fang* (Wang G et al., 2020). With the progress of modern pharmaceutical technology and the demands of patients, CXCTP has evolved into various dosage forms (Wang G et al., 2020). Nine dosage forms have been reported in the literature, including powders, pills, concentrated pills, drip pills (Rui, Xie, and Tong, 2007), tablets, granules, bagged tea, oral liquid (Hu, Jian, Wang, and Ji, 2012), and soft capsules (Gao and He, 2005). Chinese Pharmacopoeia 2020 edition includes six dosage forms: powders, pills, concentrated pills, tablets, granules, and bagged tea. In this study, we chose powders and pills of CXCTP as the subject to compare the differences in dissolution and pharmacokinetics of tetramethylpyrazine, ferulic acid, nodakenin, and isoimperatorin (TMP, FA, NK, and ISP) and discuss the scientific rationality of the classic TCM theory that “components in pills release slowly and take effect in slow-acting manner, while in powders release quickly and take effect in fast-acting way.” Powders with small particle size and large specific surface area have a fast onset of action (Xiang and Miao, 2019), while pills with slowly dissolved dispersion behavior are often used as a slow-release formulation (Zhang et al., 2017; Dong et al., 2021). Therefore, powders and pills with significant dosage form differences were selected to explore the effect of the dosage form of CXCTP on *in vivo* pharmacokinetics and *in vitro* dissolution of the four analytes.

In summary, we measured the *in vitro* dissolution of the four analytes of Chuanxiong Chatiao powders and pills and conducted an *in vivo* pharmacokinetic study with female rabbits. Based on the preparation theory of “components in pills release slowly and take effect in slow-acting manner, while in powders release quickly and take effect in fast-acting way,” we discussed the scientific rationality of the phenomenon of “Different Dosage Forms of the Same Prescription” and hoped to lay a foundation for future research on the rationality of dosage form design of Chinese medicine compound preparations.

## 2 Materials and Methods

### 2.1 Materials and Reagents

Ferulic acid control (99.94% purity, batch number MΜST-19032928), tetramethylpyrazine control (99.95% purity, batch number MΜST-19051403), nodakenin control (99.92% purity, batch number MΜST-19080408), and isoimperatorin control (99.27% purity, batch number MΜST-19032301) were all purchased from Chengdu Manster Biological Products Co., Ltd. (Chengdu, China). Formic acid was acquired from Tianjin Kemiou Chemical Reagent Co., Ltd. (Tianjin, China). Sodium heparin (150 μ/mg) was bought from Shanghai Yuanye Bio-Technology Co., Ltd. (Shanghai, China). Urethane was obtained from Shanghai Shanpu Chemical Co., Ltd. (Shanghai, China). Acetonitrile was chromatographically pure. Water was Watson’s distilled water. Other chemicals were of analytical grade and all from a commercial source. Chuanxiong Chatiao powders and pills were made in the laboratory.

Chuan Xiong (batch number 20181202), Bai Zhi (batch number 20190901), Qiang Huo (batch number 20190501), Xi Xin (batch number 20190502), Fang Feng (batch number 20191101), Jing Jie (batch number 20191101), Bo He (batch number 0190801), and Gan Cao (batch number 20191002) were all purchased from Shaanxi Xingshengde Pharmaceutical Co., Ltd. (Tongchuan, China), and all botanical identifications were authenticated by Professor Hu Benxiang (Department of Raw Medicine, Shaanxi University of Chinese Medicine, Xianyang, China). Chuan Xiong is the dried rhizome of *Ligusticum striatum* DC., family Apiaceae. Bai Zhi is the dried root of *Angelica dahurica* (Hoffm.) Benth. and Hook. f. *ex Franch. and Sav*., family Apiaceae. Qiang Huo is the dried rhizome and root of *Hansenia weberbaueriana* (Fedde ex H. Wolff) Pimenov and Kljuykov, family Apiaceae. Fang Feng is the dried roots of *Saposhnikovia divaricata* (Turcz. ex Ledeb.) Schischk., family Apiaceae. Xi Xin is the dried roots and rhizomes of *Asarum heterotropoides* F. Schmidt, family Aristolochiaceae. Jing Jie is the dried above-ground part of *Nepeta tenuifolia* Benth., family Lamiaceae. Bo He is the dried above-ground part of *Mentha canadensis* L., family Lamiaceae. Gan Cao is the dried roots and rhizomes of *Glycyrrhiza uralensis* Fisch. ex DC., family Fabaceae.

### 2.2 Preparation of Chuanxiong Chatiao Powders and Pills

Chuanxiong Chatiao powders were prepared in the laboratory according to the preparation method in the Chinese Pharmacopoeia 2020 edition. 120 g Chuan Xiong, 60 g Bai Zhi, 60 g Qiang Huo, 30 g Xi Xin, 45 g Fang Feng, 120 g Jing Jie, 240 g Bo He, and 60 g Gan Cao were weighed according to prescription quantity, put into a pulverizer, crushed into a fine powder, passed through the No. 6 sieve to remove the coarse powder, and mixed to get Chuanxiong Chatiao powders.

Chuanxiong Chatiao pills were prepared in the laboratory according to the preparation method in the Chinese Pharmacopoeia 2020 edition. 120 g Chuan Xiong, 60 g Bai Zhi, 60 g Qiang Huo, 30 g Xi Xin, 45 g Fang Feng, 120 g Jing Jie, 240 g Bo He, and 60 g Gan Cao were weighed according to prescription quantity, crushed into a fine powder. 5% mixed herbs powder of the prescription amount was weighed, and the appropriate amount of distilled water was added to obtain wet mass in the state of “holding the wet mass in your hand and it will form a cluster, and lightly pressing and it will disperse,” squeezing the wet mass through the No. 1 sieve to make the medicine molds. Then, the No. 2 sieve was used to remove the small medicine molds and the molds that stuck together, and water and fine powder were added until the medicine molds were formed. After forming the right size of medicine molds, the pills were made using water pills (alternately adding water and fine powder so that the pellets gradually increase in size), and the dry drug powder was used to cover the surface of the medicine molds. During the drying process, the temperature of the vacuum drying oven was set to 60°C; the pills were often turned over to avoid uneven drying and taken out and cooled after 4 h. Those with round shapes, uniform sizes, and weight differences and meeting the quality requirements were selected to obtain Chuanxiong Chatiao pills.

We used the external standard method to calculate the average content of TMP, FA, NK, and ISP in Chuanxiong Chatiao powders (0.1191, 0.3244, 1.0416, and 1.1759 mg/g) and Chuanxiong Chatiao pills (0.1228, 0.3246, 0.6615, and 0.7865 mg/g), and the results showed that the RSDs of the four ingredients of powders and pills were within 5.00% in three batches of samples.

### 2.3 Animals, Drug Administration, and Sampling of *In vivo* Pharmacokinetic Study

Six domestic rabbits (female, weighted 2.35 ± 0.34 kg) were purchased from Chengdu Da Shuo Biotechnology Co., Ltd. (Chengdu, China). All rabbits were fed in the animal laboratory of the School of Pharmacy, South Campus of the Shaanxi University of Chinese Medicine, and the environment was maintained under specific conditions (23 ± 2°C, 55 ± 10% relative humidity, and 12 h light/dark cycle). After 7 days, they were prohibited with water and food for 12 h before drug administration. Animal experiments were reviewed and approved by the Animal Ethics Committee of the Shaanxi University of Chinese Medicine, and the experimental animal license was SCXK (Sichuan) 2020–030.

For the pharmacokinetic study, six female rabbits were divided into two groups (*n* = 3) using the random number table method, and the maximum dose method was used to determine the dose of rabbits. Chuanxiong Chatiao powders and pills were administered by operation at a single dose of 9.85 g/kg (17.59 times the clinical dosage of humans), respectively. A Y-shaped indwelling needle was embedded in the distal end of the auricular artery of each rabbit, secured with medical tape. Then, the heparin cap of the indwelling needle was unscrewed, and 1.0 ml of diluted sodium heparin solution was injected slowly, sealed, and capped with the heparin cap. Elizabethan collars were put around the neck of rabbits to prevent them from pulling out the indwelling needle. Subsequently, 25% urethane solution was injected into the abdominal cavity of rabbits at a dose of 4.0 ml/kg. After anesthesia, the hair near the stomach was removed and disinfected with iodophor; the cortex and muscle layer of the stomach were divided with a scalpel; part of the stomach was moved out; a small incision of 1.0 cm was cut to give drugs; surgical sutures were used to suture the stomach, muscle layer, and cortex; and then erythromycin ointment was applied to incision with a cotton swab. At 5, 15, 30, 45, 60, 90, 120, 150, 180, 240, 360, 480, 720, and 1,440 min after administration, blood samples (1.5 ml) were taken from the indwelling needle, placed in anticoagulated EP tubes, centrifuged at 4°C and 4,000 rpm for 10 min, and stored at –20°C until analysis.

### 2.4 Plasma Sample Processing

150 μL of plasma samples was taken, and 500 μL of methanol was added for extraction. After vortexing for 3 mins, the mixture was centrifuged at 13,500 rpm for 10 min. The supernatants were blown dry with nitrogen, re-dissolved with 150 μL of methanol, vortexed for 3 mins, sonicated for 5 mins, and centrifuged at 13,500 rpm for 10 min. The final supernatants were used for LC-MS/MS analysis.

### 2.5 Analysis of TMP, FA, NK, and ISP in Dissolved Solutions and Rabbit Plasma Samples

We used 20 A ΜPLC- Q-trap 4500 MS (Shimadzu, Japan, SCIEX, United States) to analyze the four components of the dissolved solutions and rabbit plasma samples. TMP, FA, NK, and ISP were scanned with an electrospray ionization source (ESI) in positive ion detection mode. The multiple reaction monitoring (MRM) mode was applied for quantitative analysis. The instrument parameters included an ion spray voltage of 5.5 kV, capillary temperature of 500°C, GS1 of 60 psi, and GS2 of 50 psi. The MS parameters of the four analytes are shown in Table 2. Data analysis was conducted with Analyst version 1.6.2 (Applied Biosystems/MDS SCIEX).

**TABLE 2:** Mass spectrometry conditions of tetramethylpyrazine, ferulic acid, nodakenin, and isoimperatorin.

Component	Retention time (min)	Parent ion (m/z)	Daughter ion (m/z)	DP (V)	CE (V)
Tetramethylpyrazine	1.13	137.20	55.00	68.00	30.00
Ferulic acid	1.28	195.00	177.10	54.00	14.00
Nodakenin	1.00	409.30	247.00	100.00	18.00
Isoimperatorin	5.82	271.10	203.00	99.00	17.00

Four analytes were separated on ACQMITY MPLC®BEH C_18_ (1.7 μm, 2.1 × 100 mm) at 30°C with a flow rate of 0.3 ml/min. The mobile phase consisted of acetonitrile (A) and 0.1% formic acid water (B) with gradient elution. For quantitative determination of the four ingredients, the gradient elution program was as follows: 0.0–0.1 min, 30% A; 0.1–2.6 min, 30%–50% A; 2.6–3.0 min, 50%–61% A; 3.0–7.5 min, 61% A; 7.5–10.0 min, 61%–30% A; and 10.1 min, stop. The injection volume of samples was 2 μL.

### 2.6 *In Vitro* Solubility Study

Three portions of Chuanxiong Chatiao powders and pills were weighed separately, each with 6.00 g. Dissolution of Chuanxiong Chatiao powders and pills was measured according to the paddle method in the Chinese Pharmacopoeia 2020 edition. 900 ml of 0.1 mol/L aqueous hydrochloric acid solution was measured, degassed by ultrasound as the dissolution medium. The experimental temperature was 37°C, and the speed of the stirrer was 100 r/min. 4 ml of the dissolved solution was sucked at the same place at 2, 5, 15, 30, 45, 60, 90, 120, 150, 180, 210, and 240 min, respectively, as well as supplemented isothermal equal volume aqueous hydrochloric acid solution simultaneously. The dissolved solutions were filtered with 0.22 μm microporous membrane after coarse filtration. 2 μL subsequent filtrate was taken to determine the content of TMP, FA, NK, and ISP in Chuanxiong Chatiao powders and pills by LC-MS/MS. The cumulative dissolution rates of each component and plotted dissolution curves were calculated.

### 2.7 Solution Preparation of *In Vivo* Pharmacokinetic Study and *In Vitro* Solubility Study

#### 2.7.1 Preparation of Dilute Heparin Sodium and 25% Urethane Solution

0.50 g heparin sodium solid powder was weighed, added to 50 ml saline, stirred for 2 min using a clean glass rod, and then sonicated for 5 min. 25.00 g urethane was weighed, added to 100 ml distilled water, and sonicated for 10 min to get a 25% urethane solution.

#### 2.7.2 Preparation of Control Solution

Appropriate amounts of TMP, FA, NK, and ISP controls were weighed by electronic analytical balance, the four controls were placed into a 5 ml volumetric flask separately, and chromatographic methanol was added to the scale line to obtain the control stock solution with concentrations of 1.3940, 1.5460, 1.6440, and 1.3280 mg/ml. Took 0.4 ml of control stock solution of TMP, FA, and NK and 0.5 ml of ISP into a 100 ml volumetric flask, fixed the volume with methanol to the scale line to obtain mixed control solution I with concentrations of 5.5760, 6.1840, 6.5760, and 6.9100 μg/ml, placed the control stock solution and mixed control solution I in refrigerator at 4°C.

#### 2.7.3 Preparation of Test Solution

Six copies of Chuanxiong Chatiao powders and pills were weighed, 6.00 g per serving. According to the paddle method, 900 ml of 0.1 mol/L hydrochloric acid solution was taken after degassing as a dissolution medium. The experimental temperature was 37°C, and the stirring speed was 100 r/min. When the temperature of the dissolution medium reached the experimental temperature, powders or pills were put into the dissolution cup, started the intelligent dissolution tester immediately and began timing. 4 ml of the dissolved solution was taken at 4 h and filtered coarsely. Then, a refined filtration was done using a 0.22 μm microporous membrane, and the continued filtrate was taken as the test solution.

#### 2.7.4 Preparation of Negative Control Solution

The negative control solution was prepared in the laboratory with self-made Chuanxiong Chatiao powders and Chuanxiong Chatiao pills, without Chuan Xiong, Bai Zhi, Qiang Huo, and Fang Feng, according to the “preparation of test solution” method.

## 3 Results

### 3.1 Method Validation of the *In Vitro* Solubility Study

#### 3.1.1 Specificity

**FIGURE 1:**
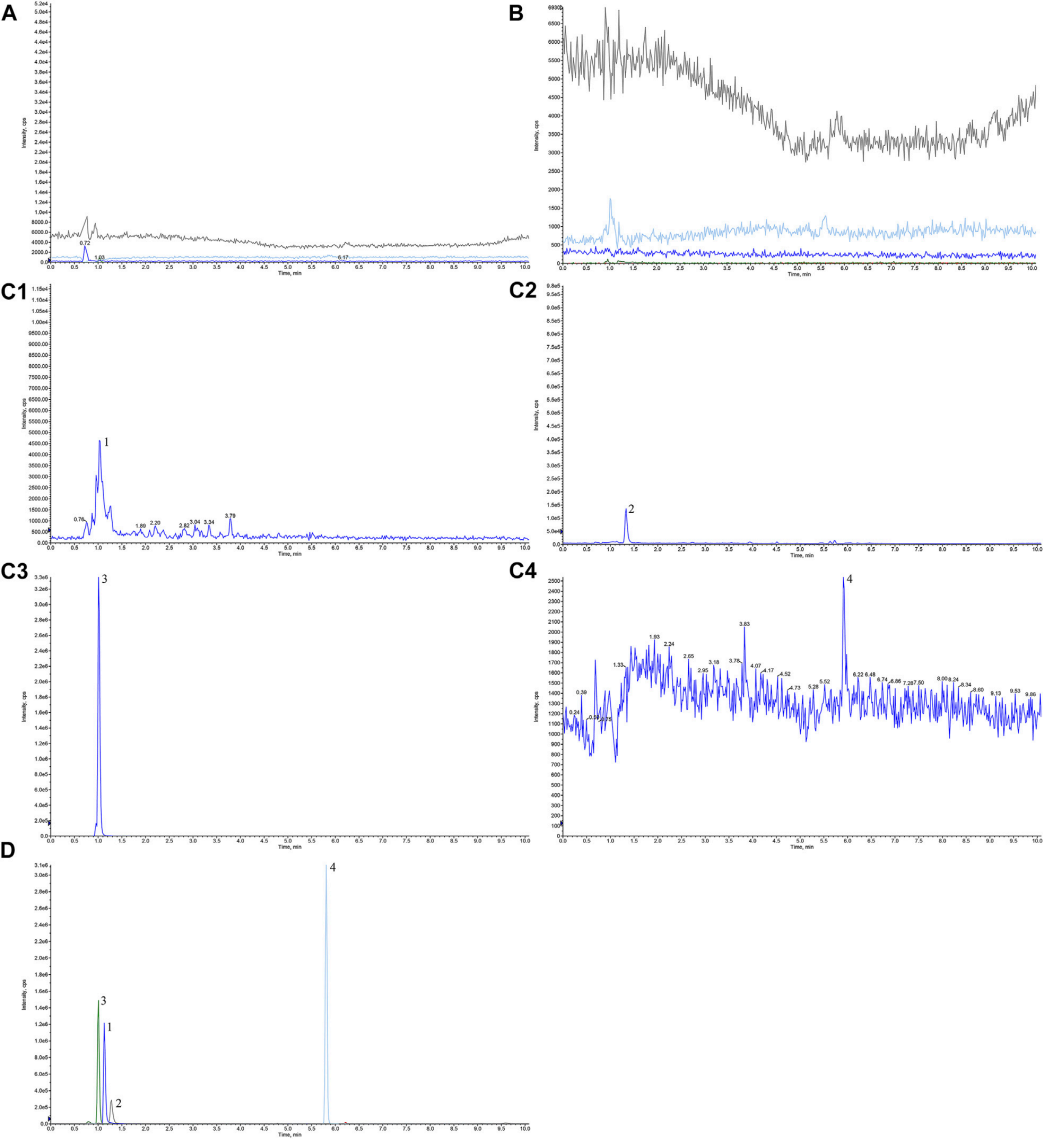
Chromatograms of tetramethylpyrazine, ferulic acid, nodakenin, and isoimperatorin in Chuanxiong Chatiao powder. **(A)** Blank solvent, **(B)** negative control solution, **(C)** test solution, **(D)** mixed control I solution. **(C1)** tetramethylpyrazine, **(C2)** ferulic acid, **(C3)** nodakenin, and **(C4)** isoimperatorin. 1, tetramethylpyrazine, 2, ferulic acid, 3, nodakenin, 4, isoimperatorin.

Appropriate amounts of blank solvent (A), negative control solution (B), test solution (C), and mixed control solution I (D) were taken, injected, and measured by LC-MS/MS. Representative chromatograms of blank solvent (A), negative control solution (B), test solution (C), and mixed control solution I (D) are shown in Figure 1. The retention times of TMP, FA, NK, and ISP were 1.03, 1.33, 1.01, and 5.92 min, respectively. The negative control showed no interference, indicating good specificity.

#### 3.1.2 Standard Curve and Linear Range

Appropriate amounts of mother liquor of TMP, NK, and ISP were pipetted into a 100 ml volumetric flask to make the mixed control solution II and diluted 1, 2, 5, 10, and 50 times with methanol. Similarly, appropriate amounts of mother liquor of FA were taken and diluted 1, 2, 10, 100, and 1,000 times with methanol, respectively. The injection volume of the above solutions was 2 μL, and the correspondent content of the four analytes was measured by LC-MS/MS. The standard curve was constructed by plotting the peak area (*Y*) *versus* the concentrations (*X*, ng/mL) of TMP, FA, NK, and ISP, and their regression equation and linear range were calculated. The results are shown in Table 3. The standard curves were linear over the concentration range of 1.1152–55.7600 ng/ml for TMP, 6.1840–6184.0000 ng/ml for FA, 1.3152–657.6000 ng/ml for NK, and 1.3820–69.1000 ng/ml for ISP. The mean linear regression equation of the standard curves was *Y*= 6,349.8 *X* + 1,667.9 with *r* = 0.9996 for TMP, *Y* = 551.17 *X*–249.6 with *r* = 0.9996 for FA, *Y* = 4,196.8 *X*–8,946 with *r* = 0.9999 for NK, and *Y* = 1,072.9 *X*–2,276.3 with *r* = 1.0000 for ISP. The lower limits of detection (LLOD) of TMP, FA, NK, and ISP were 0.5158, 1.2368, 0.3425, and 2.3000 ng/ml, and their lower limits of quantification (LLOQ) were 1.7193, 4.1227, 1.1417, and 7.6667 ng/ml, respectively.

**TABLE 3:** Regression equation and linear range of the standard curve of tetramethylpyrazine, ferulic acid, nodakenin, and isoimperatorin.

Component	Regression equation	*r*	Linear range (ng/ml)	Detection limit (ng/ml)	Quantitative limit (ng/ml)
Tetramethylpyrazine	*Y*= 6,349.80 *X* + 1,667.90	0.9996	1.1152–55.7600	0.5158	1.7193
Ferulic acid	*Y* = 551.17 *X*–249.60	0.9996	6.1840–6,184.0000	1.2368	4.1227
Nodakenin	*Y* = 4,196.80 *X*–8,946.00	0.9999	1.3152–657.6000	0.3425	1.1417
Isoimperatorin	*Y* = 1,072.90 *X*–2,276.30	1.0000	1.3820–69.1000	2.3000	7.6667

#### 3.1.3 Precision

Appropriate amount of mixed control solution was pipetted and diluted 20 times, and injection was repeated six times by LC-MS/MS. The relative standard deviations (RSDs) of peak areas were 2.63% for TMP, 1.00% for FA, 1.68% for NK, and 2.88% for ISP, respectively, all less than 3.00%.

#### 3.1.4 Reproducibility

6.00 g Chuanxiong Chatiao powders and pills each was weighed separately, and dissolution determination was repeated six times according to the paddle method. 4 ml of the dissolved solution was taken at 4 h and filtered coarsely and then refined filtration was done with 0.22 μm microporous membrane. The continued filtrate was taken for determination by LC-MS/MS. The results showed that the RSDs of mean dissolution in Chuanxiong Chatiao powder were 2.15% for TMP, 4.15% for FA, 1.14% for NK, and 4.26% for ISP, respectively, indicating good reproducibility.

#### 3.1.5 Recovery

Nine portions of Chuanxiong Chatiao powders were weighed, 6.00 g per serving, and controls were added at 50%, 100%, and 150% according to the content of the TMP, FA, NK, and ISP in the dissolved solution of powder at 4 h, respectively. Then, the recoveries were injected and calculated. Results are shown in Table 4. RSDs of recoveries were 1.0% for TMP, 1.75% for FA, 0.67% for NK, and 1.84% for ISP in Chuanxiong Chatiao powders, respectively, which were less than 3.00%, indicating good recoveries.

**TABLE 4:** Recovery of tetramethylpyrazine, ferulic acid, nodakenin, isoimperatorin in Chuanxiong Chatiao powder.

Component	Content in the sample (ng/ml)	Amount of control product added (ng/ml)	Measured value (ng/ml)	Recovery rate (%)	RSD (%)
Tetramethylpyrazine	7.44	3.70	11.16	100.54	1.07
	7.69	3.70	11.42	100.81	
	7.31	3.70	11.00	99.73	
	7.38	7.40	14.58	97.30	
	7.52	7.40	14.87	99.32	
	7.64	7.40	14.95	98.78	
	7.43	11.10	18.36	98.47	
	7.47	11.10	18.47	99.10	
	7.35	11.10	18.40	99.55	
Ferulic acid	831.81	420.51	1,251.03	99.69	1.75
	843.74	420.51	1,260.30	99.06	
	840.97	420.51	1,250.71	97.44	
	855.39	841.02	1,725.30	103.44	
	850.65	841.02	1,706.12	101.72	
	861.93	841.02	1,701.77	98.86	
	847.20	1,261.53	2,100.04	99.31	
	856.55	1,261.53	2,108.56	99.25	
	860.12	1,261.53	2,117.93	99.71	
Nodakenin	3,197.99	1,623.85	4,824.38	100.16	0.67
	3,207.41	1,623.85	4,832.48	100.07	
	3,194.63	1,623.85	4,821.52	100.19	
	3,216.41	3,247.70	6,524.01	101.86	
	3,213.75	3,247.70	6,450.85	99.67	
	3,220.24	3,247.70	6,498.51	100.94	
	3,200.33	4,871.55	8,130.23	101.20	
	3,245.17	4,871.55	8,126.18	100.20	
	3,228.74	4,871.55	8,123.80	100.48	
Isoimperatorin	14.31	7.25	21.74	102.48	1.84
	14.57	7.25	21.68	98.07	
	14.43	7.25	21.78	103.17	
	14.55	14.50	29.41	102.48	
	14.60	14.50	29.14	100.28	
	14.68	14.50	29.47	102.00	
	14.54	21.75	36.03	98.80	
	14.65	21.75	36.29	99.49	
	14.48	21.75	36.16	99.68	

#### 3.1.6 Stability

Dissolved solutions were put at 4 h of Chuanxiong Chatiao powders and pills into 50 ml conical flask separately, extracted with 30 ml of 70% methanol for 30 min at specific ultrasonic power (240 W) and room temperature (25°C), stood until stratification, and weighed. The difference was supplemented, and the solution was filtered with 0.22 μm organic membrane to obtain the filtrates for stability investigation. The filtrates were measured by LC-MS/MS at 0, 2, 4, 6, 8, 12, and 24 h, respectively. The results showed that the RSDs of the peak area values of TMP, FA, NK, and ISP in Chuanxiong Chatiao powders were 4.42%, 2.68%, 3.52%, and 3.85%, respectively, indicating that the test solution was stable within 24 h.

### 3.2 *In Vitro* Cumulative Dissolution Rate and Dissolution Curve of TMP, FA, NA, and ISP

**FIGURE 2:**
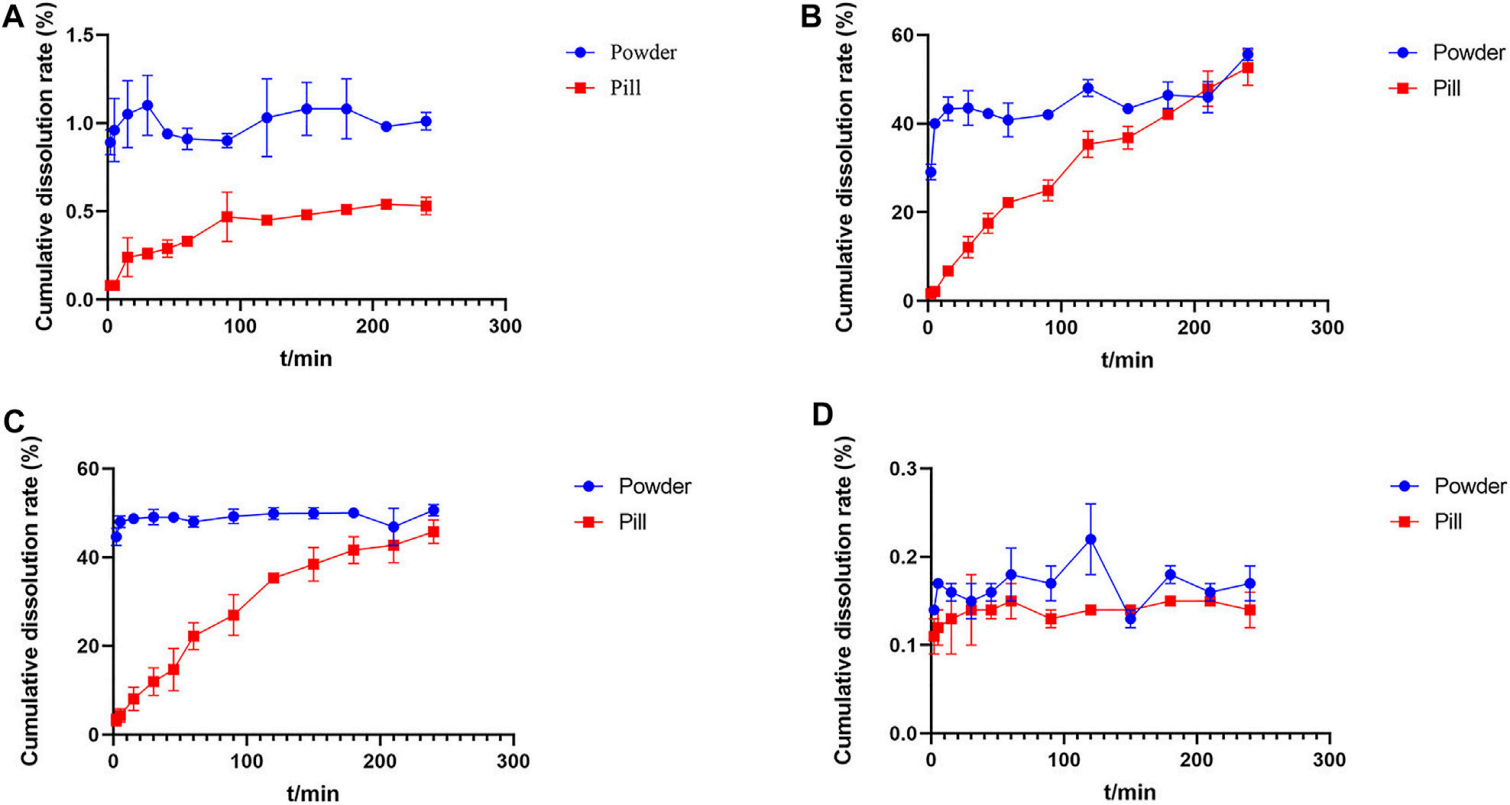
Dissolution curves of tetramethylpyrazine, ferulic acid, nodakenin, and isoimperatorin in Chuanxiong Chatiao powder and pill. **(A)** Tetramethylpyrazine, **(B)** ferulic acid, **(C)** nodakenin, and **(D)** isoimperatorin.

The cumulative dissolution rates and dissolution curves of the four analytes in Chuanxiong Chatiao powders and pills are shown in Table 5 and Figure 2, respectively. The results showed that the solubility of the four analytes in powder was significantly greater than that of the pill.

**TABLE 5:** Cumulative dissolution rates of tetramethylpyrazine, ferulic acid, nodakenin, and isoimperatorin (*n* = 3, −x ± S).

Times (min)	Powder group (ng/ml)				Pill group (ng/ml)			
	Tetramethylpyrazine	Ferulic acid	Nodakenin	Isoimperatorin	Tetramethylpyrazine	Ferulic acid	Nodakenin	Isoimperatorin
2	0.89 ± 0.07	29.08 ± 1.77	44.64 ± 1.96	0.14 ± 0.00	0.08 ± 0.00	1.69 ± 0. 52	3.34 ± 1.34	0.11 ± 0.02
5	0.96 ± 0.18	40.00 ± 0.69	48.01 ± 1.30	0.17 ± 0.00	0.08 ± 0.01	2.19 ± 0.30	4.33 ± 1.49	0.12 ± 0.02
15	1.05 ± 0.19	43.31 ± 2.65	48.72 ± 0.52	0.16 ± 0.01	0.24 ± 0.11	6.79 ± 0.86	8.06 ± 2.64	0.13 ± 0.04
30	1.10 ± 0.17	43.52 ± 3.90	49.08 ± 1.71	0.15 ± 0.02	0.26 ± 0.03	12.12 ± 2.43	11.97 ± 3.11	0.14 ± 0.04
45	0.94 ± 0.02	42.26 ± 0.60	49.01 ± 0.27	0.16 ± 0.01	0.29 ± 0.05	17.51 ± 2.25	14.68 ± 4.76	0.14 ± 0.01
60	0.91 ± 0.06	40.81 ± 3.83	48.02 ± 1.22	0.18 ± 0.03	0.33 ± 0.00	22.18 ± 0.84	22.22 ± 3.04	0.15 ± 0.02
90	0.90 ± 0.04	42.00 ± 0.23	49.21 ± 1.61	0.17 ± 0.02	0.47 ± 0.14	24.92 ± 2.35	26.96 ± 4.62	0.13 ± 0.01
120	1.03 ± 0.22	48.02 ± 1.92	49.85 ± 1.34	0.22 ± 0.04	0.45 ± 0.00	35.31 ± 2.96	35.34 ± 0.56	0.14 ± 0.00
150	1.08 ± 0.15	43.37 ± 1.00	49.94 ± 1.24	0.13 ± 0.01	0.48 ± 0.01	36.83 ± 2.53	38.43 ± 3.82	0.14 ± 0.00
180	1.08 ± 0.17	46.41 ± 3.00	50.02 ± 0.80	0.18 ± 0.01	0.51 ± 0.01	42.07 ± 0.29	41.65 ± 3.04	0.15 ± 0.00
210	0.98 ± 0.02	45.96 ± 3.50	46.89 ± 4.23	0.16 ± 0.01	0.54 ± 0.01	47.88 ± 4.01	42.73 ± 3.97	0.15 ± 0.00
240	1.01 ± 0.05	55.60 ± 1.35	50.62 ± 1.24	0.17 ± 0.02	0.53 ± 0.05	52.64 ± 3.97	45.76 ± 2.68	0.14 ± 0.02

The cumulative dissolution rate of TMP in powder reached the maximum at 30 min and tended to plateau after 120 min, which was around 1.00% from 120 to 240 min without excessive fluctuations. In contrast, the dissolution of TMP in pill was small and slow, gradually increasing with time. Meanwhile, the cumulative dissolution rate of TMP in both powder and pill was very low, which might be related to the slightly water-soluble nature of TMP and the reaction with an acidic dissolution medium. The cumulative dissolution rate of FA in powder did not vary significantly and reached about 40.00% from the second point of sampling, indicating that FA was polar and easily soluble in aqueous solvents. The dissolution of FA in pill increased with time and reached the maximum dissolution at 210 min, then showing a decreasing trend. The dissolution of NK in powder was rapidly dissolved at the first point of sampling, and the cumulative dissolution rate of each sampling point varied a little within 4 h, while the dissolution of NK in pill showed an upward trend with time. The dissolution of ISP in both powder and pill was very low, which may be related to its low solubility.

### 3.3 Method Validation of the *In Vivo* Pharmacokinetic Study

#### 3.3.1 Specificity

**FIGURE 3:**
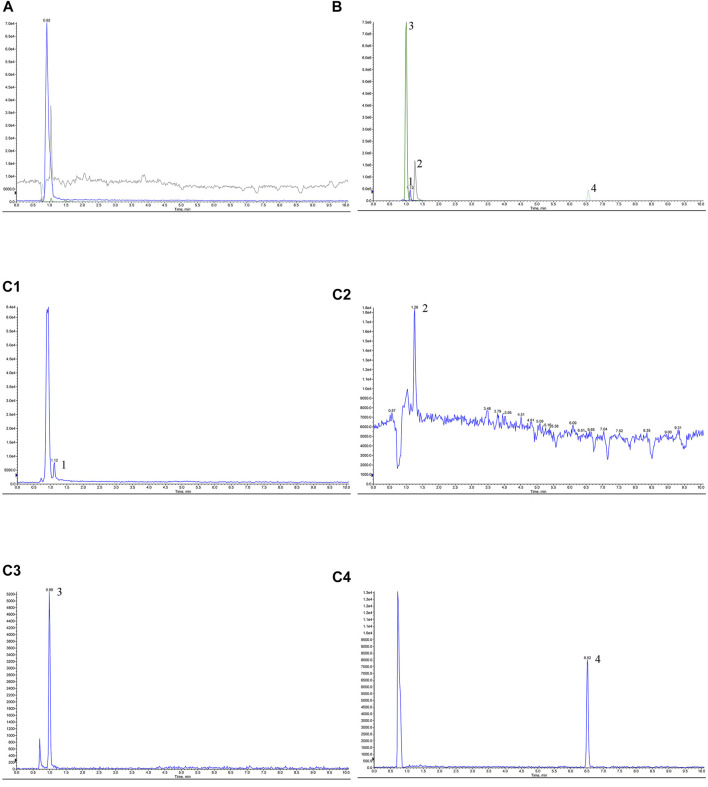
Chromatograms of tetramethylpyrazine, ferulic acid, nodakenin, and isoimperatorin of Chuanxiong Chatiao powder in rabbit plasma. **(A)** Blank plasma, **(B)** control + blank plasma, and **(C)** post-administration plasma. **(C1)** Tetramethylpyrazine, **(C2)** ferulic acid, **(C3)** nodakenin, **(C4)** isoimperatorin. 1, tetramethylpyrazine, 2, ferulic acid, 3, nodakenin, 4, isoimperatorin.

Appropriate amounts of blank plasma (A), control + blank plasma (B), and post-administration plasma (C) were taken. Those plasma samples were processed according to “Plasma Samples Processing.” Then, the samples were injected. Representative chromatograms of blank plasma (A), control + blank plasma (B), and post-administration plasma (C) are shown in Figure 3. The retention times of TMP, FA, NK, and ISP were 1.12, 1.26, 0.99, and 6.52 min, respectively, and the blank plasma showed no chromatographic interference at the retention time of TMP, FA, NK, and ISP, indicating good specificity.

#### 3.3.2 Standard Curve and Linear Range

150 μL of blank plasma was taken, and 50 μL of the mixed control solution I was added at different dilutions times separately. The above samples were processed according to “Plasma Samples Processing,” and the correspondent content of the four analytes was measured by LC-MS/MS. The standard curve was constructed by plotting the peak area (*Y*) *versus* the concentrations (*X*, ng/mL) of TMP, FA, NK, and ISP in rabbit plasma, and the regression equation and their linear range were calculated. The results are presented in Table 6. The standard curves were linear over the concentration range of 1.7425–69.7000 ng/ml for TMP, 9.6625–4,831.2500 ng/ml for FA, 1.0275–205.5000 ng/ml for NK, and 3.3200–166.000 ng/ml for ISP. The mean linear regression equation of the standard curves was *Y* = 542.07 *X* + 4,119.4 with *r* = 0.9994 for TMP, *Y* = 1,449.2 *X*–36,481 with *r* = 0.9999 for FA, *Y* = 4,097.9 *X*–3,191.4 with *r* = 0.9998 for NK, and *Y* = 859.24 *X*–556.05 with *r* = 0.9996 for ISP. The lower limits of detection (LLOD) of TMP, FA, NK, and ISP were 0.5158, 1.2368, 0.3425 and 2.3000 ng/ml, and their lower limits of quantification (LLOQ) were 1.7193, 4.1227, 1.1417, and 7.6667 ng/ml, respectively.

**TABLE 6:** Regression equation and linear range of the standard curve of tetramethylpyrazine, ferulic acid, nodakenin, and isoimperatorin in rabbit plasma.

Component	Regression equation	*r*	Linear range (ng/ml)	Detection limit (ng/ml)	Quantitative limit (ng/ml)
Tetramethylpyrazine	*Y* = 542.07 *X*+ 4,119.40	0.9994	1.7425–69.7000	0.5158	1.7193
Ferulic acid	*Y* = 1,449.20 *X*–36,481.00	0.9999	9.6625–4,831.2500	1.2368	4.1227
Nodakenin	*Y* = 4,097.90 *X*–3,191.40	0.9998	1.0275–205.5000	0.3425	1.1417
Isoimperatorin	*Y* = 859.24 *X*–556.05	0.9996	3.3200–166.0000	2.3000	7.6667

#### 3.3.3 Precision

The quality control samples of the following high, medium, and low concentrations were prepared: 2788.000, 1394.000, 13.940 ng/ml of TMP; 3,865.000, 1,932.500, 19.325 ng/ml of FA; 1027.500, 318.525, 3.185 ng/ml of NK; and 1328.000, 46.480, 23.240 ng/ml of ISP. Six QC samples of each of the four analytes were prepared in parallel for each concentration, processed those samples according to “Plasma Samples Processing”, then determined those samples by LC-MS/MS within 24 h and calculated the RSD of intra-day precision. The RSD of inter-day precision was calculated with continuous measurement for 3 days. Results of intra- and inter-day precision of TMP, FA, NK, and ISP are summarized in Table 7. The RSDs of intra-day precision (coefficient of variation, CV) were less than 2.14% for TMP, 2.30% for FA, 2.81% for NK, and 3.02% for ISP, and RSDs of inter-day precision (CV) were within 3.25% for TMP, 4.85% for FA, 3.67% for NK, and 3.49% for ISP. Therefore, this method indicated good precision.

**TABLE 7:** Intra-day and inter-day precision of tetramethylpyrazine, ferulic acid, nodakenin, and isoimperatorin in rabbit plasma (*n* = 6).

Component	Concentration (ng/ml)	Intra-day RSD (%)	Inter-day RSD (%)
Tetramethylpyrazine	2,788.000	1.14	2.50
	1,394.000	1.98	2.55
	13.940	2.14	3.25
Ferulic acid	3,865.000	2.30	3.07
	1,932.500	2.07	2.24
	19.325	2.12	4.85
Nodakenin	1,027.500	2.40	3.01
	318.525	2.47	3.58
	3.18525	2.81	3.67
Isoimperatorin	1,328.000	2.40	2.91
	46.480	2.68	2.63
	23.240	3.02	3.49

#### 3.3.4 Recovery and Matrix Effect

150 μL blank plasma was taken and processed according to “Plasma Samples Processing.” Appropriate amounts of control solutions of TMP, FA, NK, and ISP were added with three different concentrations of high, medium, and low, blew dry with nitrogen, re-dissolved with methanol 150 μL, 6 in parallel for each concentration, and injected. The peak area was recorded as S1. Another 150 μL of blank plasma was taken, and appropriate amounts of control solutions of TMP, FA, NK, and ISP were added with three different concentrations of high, medium, and low. The samples were processed according to “Plasma Samples Processing,” six in parallel for each concentration, blew dry with nitrogen, re-dissolved with methanol 150 μL, and injected. The peak area was recorded as S2. Extraction recovery rate (RS) was equal to S2 divided by S1, as shown in Table 8. Extraction recoveries for TMP, FA, NK, and ISP in rabbit plasma ranged from 85.00% to 115.00%. Appropriate amounts of control solutions of TMP, FA, NK, and ISP were taken with three different concentrations of high, medium, and low, and the samples were processed according to “Plasma Samples Processing,” blew dry with nitrogen, re-dissolved with methanol 150 μL, and injected. The peak area was recorded as S3. TS was equal to S2 divided by S3, TS was between 85% and 115%, and there was no obvious matrix effect.

**TABLE 8:** Extraction recovery of tetramethylpyrazine, ferulic acid, nodakenin, and isoimperatorin in rabbit plasma (*n* = 6).

Component	Concentration (ng/ml)	Recovery rate (%)	Concentration (ng/ml)	Recovery rate (%)	Concentration (ng/ml)	Recovery rate (%)
Tetramethylpyrazine	2788.000	102.20	1394.000	113.98	13.940	88.92
Ferulic acid	3865.000	95.84	1932.500	95.41	19.325	104.58
Nodakenin	1027.500	94.51	318.525	87.56	3.185	85.34
Isoimperatorin	1328.000	87.27	46.480	85.99	23.240	85.34

#### 3.3.5 Stability

Quality control samples of high, medium, and low concentrations, six in parallel for each concentration, were taken, their short-term stability was examined under room temperature, and measurement was repeated six times within 24 h by LC-MS/MS. The stabilities of TMP, FA, NK, and ISP are summarized in Table 9. The RSDs of short-term stability were less than 4.50% for TMP, 3.83% for FA, 4.22% for NK, and 4.82% for ISP. Therefore, these four components were stable at room temperature within 24 h.

**TABLE 9:** Stability of tetramethylpyrazine, ferulic acid, nodakenin, and isoimperatorin in rabbit plasma (*n* = 6).

Component	Concentration (ng/ml)	Short-term stability RSD (%)
Tetramethylpyrazine	2,788.000	2.97
	1,394.000	1.82
	13.940	4.50
Ferulic acid	3,865.000	2.68
	1,932.500	2.91
	19.325	3.83
Nodakenin	1,027.500	3.79
	318.525	4.22
	3.18525	3.75
Isoimperatorin	1,328.000	2.59
	46.480	3.88
	23.240	4.82

### 3.4 Mean Plasma Concentration-Time Curves and Pharmacokinetic Parameters of TMP, FA, NA, and ISP

**FIGURE 4:**
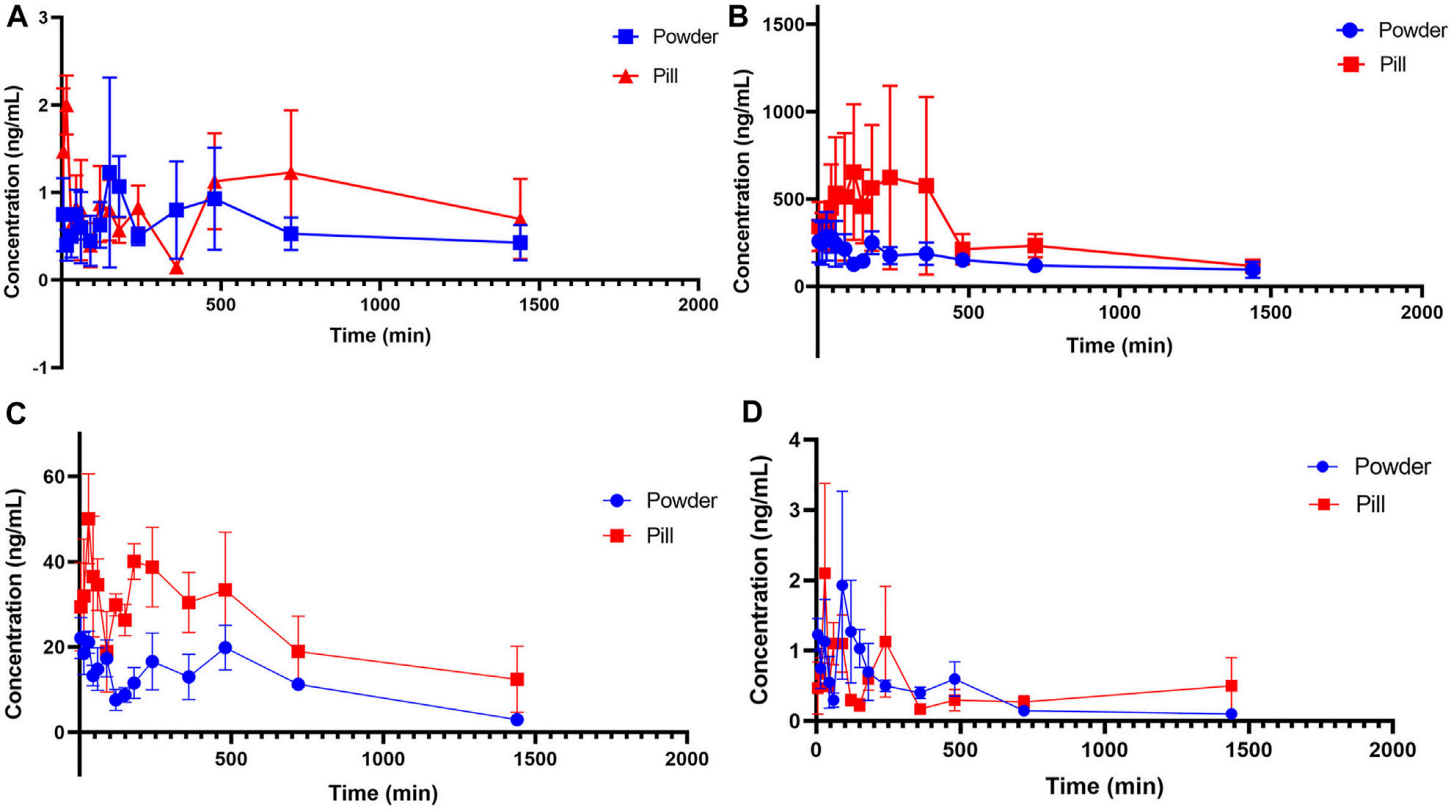
Mean plasma concentration-time profiles of tetramethylpyrazine, ferulic acid, nodakenin, and isoimperatorin in the powder and pill groups. **(A)** Tetramethylpyrazine, **(B)** ferulic acid, **(C)** nodakenin, and **(D)** isoimperatorin.

The validated simultaneous LC-MS/MS analytical method was successfully applied to the plasma pharmacokinetic study of TMP, FA, NK, and ISP in Chuanxiong Chatiao powders and pills after surgical administration (9.85 g/kg) in female domestic rabbits. The mean plasma concentration-time data of the four analytes in powder and pill are shown in Table 10, and the mean plasma concentration-time curves are presented in Figure 4. The pharmacokinetic parameters of the four analytes processed by the non-compartmental model are listed in Table 11.

**TABLE 10:** Mean plasma concentration-time data of TMP, FA, NK, and ISP in the powder and pill groups (*n* = 3,−x ± SE).

Times (min)	Powder group (ng/ml)				Pill group (ng/ml)			
	Tetramethylpyrazine	Ferulic acid	Nodakenin	Isoimperatorin	Tetramethylpyrazine	Ferulic acid	Nodakenin	Isoimperatorin
5	0.75 ± 0.42	259.07 ± 120.79	22.07 ± 4.82	1.23 ± 0.23	1.47 ± 0.72	343.17 ± 140.30	29.40 ± 10.36	0.47 ± 0.37
15	0.40 ± 0.18	247.30 ± 121.94	18.47 ± 4.94	0.75 ± 0.29	2.00 ± 0.34	314.60 ± 106.36	31.97 ± 13.34	0.67 ± 0.23
30	0.50 ± 0.24	286.73 ± 138.47	21.13 ± 2.60	1.13 ± 0.60	0.63 ± 0.13	365.77 ± 116.84	50.07 ± 10.52	2.10 ± 1.28
45	0.75 ± 0.28	284.63 ± 90.45	13.27 ± 2.39	0.55 ± 0.37	0.83 ± 0.37	449.57 ± 249.11	36.47 ± 14.17	0.50 ± 0.08
60	0.60 ± 0.41	243.73 ± 131.13	14.8 ± 5.01	0.30 ± 0.10	0.80 ± 0.57	533.23 ± 321.95	34.60 ± 6.04	1.10 ± 0.30
90	0.45 ± 0.29	213.93 ± 84.55	17.30 ± 4.33	1.93 ± 1.34	0.40 ± 0.25	512.13 ± 364.76	18.90 ± 9.49	1.10 ± 0.41
120	0.63 ± 0.26	127.60 ± 26.37	7.57 ± 2.43	1.27 ± 0.73	0.87 ± 0.43	654.70 ± 387.64	29.87 ± 2.61	0.30 ± 0.08
150	1.23 ± 1.08	145.07 ± 5.88	8.73 ± 1.70	1.03 ± 0.27	0.80 ± 0.35	457.73 ± 212.31	26.33 ± 3.68	0.23 ± 0.09
180	1.07 ± 0.35	250.23 ± 65.40	11.53 ± 3.63	0.70 ± 0.40	0.57 ± 0.15	564.33 ± 359.83	40.03 ± 4.18	0.60 ± 0.16
240	0.50 ± 0.10	176.87 ± 48.15	16.60 ± 6.68	0.50 ± 0.08	0.83 ± 0.25	623.13 ± 525.94	38.73 ± 9.29	1.13 ± 0.79
360	0.80 ± 0.56	188.07 ± 64.80	13.00 ± 5.33	0.40 ± 0.08	0.15 ± 0.04	576.70 ± 508.83	30.43 ± 7.08	0.17 ± 0.03
480	0.93 ± 0.58	151.50 ± 20.45	19.83 ± 5.26	0.60 ± 0.24	1.13 ± 0.55	213.17 ± 86.83	33.40 ± 13.54	0.03 ± 0.15
720	0.53 ± 0.19	120.20 ± 28.10	11.27 ± 0.88	0.15 ± 0.04	1.23 ± 0.71	233.73 ± 66.95	18.93 ± 8.32	0.27 ± 0.09
1440	0.43 ± 0.20	96.03 ± 46.30	2.97 ± 0.27	0.10 ± 0.00	0.70 ± 0.46	116.27 ± 18.57	12.40 ± 7.75	0.50 ± 0.40

**TABLE 11:** Pharmacokinetic parameters of tetramethylpyrazine, ferulic acid, nodakenin, and isoimperatorin in powder group and pill group (*n* = 3,−x ± S).

Pharmacokinetic parameters	Powder group (ng/ml)				Pill group (ng/ml)			
	Tetramethylpyrazine	Ferulic acid	Nodakenin	Isoimperatorin	Tetramethylpyrazine	Ferulic acid	Nodakenin	Isoimperatorin
AUC_0-t_ (ng/L*h)	21.30 ± 4.27	3676.20 ± 1,113.23	338.17 ± 153.41	8.34 ± 2.45	22.37 ± 19.27	8,668.75 ± 6,388.07	697.92 ± 115.84	9.83 ± 9.05
AUC_0-∞_(ng/L*h)	39.94 ± 17.10	5,766.23 ± 1,929.36	601.98 ± 191.55	9.91 ± 1.98	55.08 ± 69.44	9,727.90 ± 5,946.39	1,667.63 ± 1,720.98	14.64 ± 15.07
MRT_0-t_(h)	12.01 ± 2.87	10.98 ± 0.52	9.27 ± 1.08	5.14 ± 0.98	11.08 ± 1.45	8.75 ± 1.69	9.37 ± 1.89	9.42 ± 2.70
MRT_0-∞_(h)	26.00 ± 15.78	18.27 ± 12.62	10.74 ± 8.95	9.34 ± 0.52	14.29 ± 7.19	15.53 ± 6.89	39.72 ± 52.30	17.26 ± 8.11
*t*_1/2_ (h)	15.29 ± 7.57	7.66 ± 4.80	7.09 ± 5.31	8.48 ± 1.77	8.78 ± 3.87	8.42 ± 4.39	27.03 ± 37.60	12.83 ± 8.95
*T*_max_ (h)	12.83 ± 10.77	1.33 ± 1.44	0.22 ± 0.24	1.33 ± 0.76	3.75 ± 3.93	3.17 ± 0.76	4.17 ± 3.75	0.67 ± 0.29
*C*_max_ (ng/L)	2.13 ± 1.17	354.83 ± 203.60	31.93 ± 13.06	3.20 ± 1.23	2.30 ± 2.11	1124.30 ± 667.80	56.03 ± 12.96	2.23 ± 2.07

From the mean plasma concentration-time curves, we could know that the blood concentration of TMP in the powder group was generally higher than that in the pill group between 130 and 480 min, while the blood concentration of TMP in the powder group was lower than that in the pill group around 240 min. In the first 15 min, the ISP in the powder group was released faster and the blood concentration was higher than that in the pill group. From 80 to 180 min and 360 to 630 min, the blood concentration of ISP in powder was higher than that of the pill. The blood concentrations of FA and NK in the powder group were generally lower than those in the pill group. *T*_max_ (h) of FA and NK in the powder group was 0.420 and 0.053 times those of the pill group, and *t*_1/2_ (h) of FA, NK, and ISP of the powder group was 0.910, 0.262, and 0.661 times that of the pill group, respectively. *C*_max_ of NK in the powder group was 1.435 times that of the pill group. MRT_0-t_ (h) of NK and ISP in the powder group was 0.989 and 0.546 times that of the pill group, and MRT_0-∞_ (h) of NK and ISP in the powder group was 0.270 and 0.541 times that of the pill group. All AUC_0-t_ (ng/L*h) and AUC_0-∞_(ng/L*h) in the powder group were lower than those of the pill group. However, *T*_max_ (h) and *t*_1/2_ (h) of TMP, some other pharmacokinetic parameters, and blood concentrations of the four analytes were not in accord with the classic TCM theory that “components in pills release slowly and take effect in slow-acting manner, while in powders release quickly and take effect in fast-acting way.”

## 4 Discussion

The *in vitro* dissolution experiment and *in vivo* pharmacokinetic study of the powders and pills of Chuanxiong Chatiao prescription revealed that two different dosage forms had a complex release behavior. On the basis of the traditional dosage form theory of “components in pills release slowly and take effect in slow-acting manner, while in powders release quickly and take effect in fast-acting way,” the *in vitro* dissolution behavior of powders should be faster than that of pills, and the ideal *T*_max_ (h), *t*_1/2_ (h), MRT_0-t_, and MRT_0-∞_ (h) of pills should be longer than powders while *C*_max_ (ng/L), AUC_0-t_ (ng/L*h), and AUC_0-∞_(ng/L*h) of pills will be less than powders. *In vitro* studies indicated that the dissolution rates of TMP, FA, NK, and ISP in the powder group were greater than that in the pill group and could reach the plateau quickly, while slower in the pill group. *In vivo* pharmacokinetic studies showed that *T*_max_ (h) of FA and NK in the powder group was 0.420 and 0.053 times that of the pill group and *t*_1/2_ (h) of FA, NK, and ISP of the powder group was 0.910, 0.262, and 0.661 times that of the pill group, respectively. The results of *in vitro* dissolution experiment and some of the *in vivo* pharmacokinetic parameters could prove the scientific rationality of the classic TCM theory that “components in pills release slowly and take effect in slow-acting manner, while in powders release quickly and take effect in fast-acting way.” However, *T*_max_ (h) and *t*_1/2_ (h) of TMP and some other pharmacokinetic parameters of TMP, FA, NK, and ISP were not in accord with this theory. It is possible that their drug release pattern is highly correlated with their own physicochemical properties and drug interactions, and it could not be excluded that the results were not accurate enough due to the small sample size of experimental animals, which will be improved by increasing the sample size of animals and experimental repetitions.

In the metabolic process of TMP, the studies by Xiaohua et al. discovered that TMP could induce the drug metabolizing enzyme CYP3A4 by activating PXR receptors and the activity of CYP3A4 could be ascended in a dose-dependent manner (Wu, Zhu, Zhang, and Dai, 2017). Compared with the pill group, TMP in the powder group may have a stronger induction of CYP3A4 at the higher dissolution (Wu et al., 2017), which may contribute to faster metabolism of TMP and express a slower increase of TMP in blood per unit time in the powder group. The above discussion hopes to explain some pharmacokinetic parameters of TMP that did not meet expectations, such as longer *T*_max_, *t*_1/2_, MRT_0-t_, and MRT_0-∞_ and lower *C*_max_ in the powder group.

The cellular transport mechanisms of FA mainly include passive diffusion and carrier-mediated transport (Liang et al., 2015), and Liang Xinli et al. suggested that carrier transport might exist when FA was present at high concentrations (Liang et al., 2012). Compared to the pill group, when FA in the powder group dissolved with high concentration, other water-soluble ingredients would dissolve in large quantities. The water-soluble ingredients might compete with FA for binding to transporter protein, which could affect its absorption and lead to lower *C*_max_ in the powder group.

In 2010, Feng et al. suggested that the compatibility of TMP and FA caused interaction in pharmacokinetics and their *t*_1/2_ and MRT were prolonged (Feng et al., 2010, Zhang, F., Chen, and Xu, 2010). In 2012, they found that FA could prolong TMP’s action time in rats (Feng, Zhang, Zhang, Chen, and Xu, 2012), and in 2014, they discovered that TMP expanded the action time of FA and increased its absorption in rats (Feng, Zhang, and Xu, 2014). The pharmacokinetic interaction between TMP and FA may be an important reason why some of their pharmacokinetic parameters in the powder and pill groups did not conform to the classic TCM theory that “components in pills release slowly and take effect in slow-acting manner, while in powders release quickly and take effect in fast-acting way.”

Zhang et al. showed that NK was completely converted to nodakenetin in the incubated system of human intestinal flora within 16 h and nodakenetin had no significant reduction after longer incubation (Zhang and Yang, 2009). NK is a compound that is not readily absorbed, and its absorption and transport show passive diffusion characteristics (Yang, Zhang, and Wu, 2009). From the above discussion, we could not get a reasonable explanation for the lower *C*_max_ of NK in the powder group and speculated that there might exist absorption-transport interaction of NK, which caused the lower *C*_max_ in the powder group.

ISP has good fat solubility and high permeability, and its drug release behavior may be closely related to its physical and chemical properties. ISP could be enzymatically activated by CYP2B6 and was a characterized mechanism-based inactivator of CYP2B6 (Cao et al., 2015). Compared with pills, powders might have a stronger inhibitory effect on CYP2B6 at higher concentrations, resulting in a greater reduction in the metabolic rate of ISP and showing prolonged *T*_max_. On the contrary, ISP had a strong inhibitory effect on the OAT3 transporter (Wang Z et al., 2020), which was an important channel for compounds to enter the kidney. Compared to the pill group, the higher concentration of ISP in the powder group might have a stronger inhibitory effect on the OAT3 transporter, which might bring about slower elimination and longer *T*_max_ of ISP in the powder group.

It is noteworthy that the compatibility of traditional Chinese medicine has a significant effect on the pharmacokinetic parameters of key components. Yu and Gao (2003) showed that the compatibility of Chuan Xiong with Dan Shen could result in slower absorption and lower bioavailability, and when Chuan Xiong combined with Dang Gui, Bai Shao, Shu Di, Hong Hua, and Tao Ren, the blood concentration of TMP reduced. Mi et al (2020) studied the pharmacokinetics of TMP and FA in the rat model of the blood–stasis migraine with different concentrations of gastrodin and gastrodigenin. Compared with the control group without gastrodin and gastrodigenin, they found that the *t*_1/2_, MRT, *C*_max_, and AUC_0–∞_ of TMP and FA were all increased in the experimental groups with different concentrations of gastrodin and gastrodigenin. Meanwhile, different concentrations of gastrodin and gastrodigenin unequally impacted the pharmacokinetics of TMP and FA. Shan et al. studied the metabolism-based synergy of total coumarin extract of Bai Zhi and TMP on migraine in rats. Their study showed that Bai Zhi could increase the plasma concentration of TMP by inhibiting its metabolism through interfering with CYP450s, which proved that pharmacokinetic synergistic action indeed existed in combination therapy with total coumarin extract of Bai Zhi and TMP (Feng et al., 2018).

When the dosage forms of prescription are different, the interaction between ingredients may be different because the dissolution of ingredients varies greatly. In our study, the dissolution of the four analytes of Chuanxiong Chatiao powder was significantly faster than that of pill. This phenomenon might have a great impact on their pharmacokinetic parameters, which might be the main reason for the lower AUC and blood concentrations of the four analytes in the powder group.

For *in vitro* dissolution experiment, the dissolution of ingredients in the powder and pill group was measured by UHPLC initially. However, it was found that we still could not detect the content of TMP and ISP at some sampling points as well as asarinin in the pill group even if the dose increased from 4.00 to 10.00 g. Therefore, the content measure method was changed to LC-MS/MS. In contrast, the content of asarinin 97 was still very small after switching the content measure method. Pulegone is a volatile ingredient whose content is mostly determined by gas chromatography (Cheng et al., 2021). Considering all the above factors, we chose TMP, FA, NK, and ISP as index ingredients of dissolution and pharmacokinetics.

In conclusion, *in vitro* dissolution experiment could reflect the drug release characteristics of the traditional dosage forms of pills and powders. Some of the *in vivo* pharmacokinetic parameters could also explain the classic TCM theory that “components in pills release slowly and take effect in slow-acting manner, while in powders release quickly and take effect in fast-acting way.” However, this theory could not fully explain some of the undesirable pharmacokinetic parameters of key components such as TMP and FA. This phenomenon suggests the complicacy of the dosage form design of Chinese medicine compound formulas and prompts that we should pay more attention to the rationality of the phenomenon of “Different Dosage Forms of the Same Prescription.” We will further deepen our understanding of this phenomenon by evaluating the consistency of the pharmacology and pharmacodynamics of different dosage forms of Chuanxiong Chatiao prescription.

## Data Availability

The original contributions presented in the study are included in the article/supplementary material, further inquiries can be directed to the corresponding authors.
